# Detection and Genomic Characterization of Novel Respiratory Viruses in US and Mexican Cattle Farms

**DOI:** 10.1155/tbed/3247802

**Published:** 2026-05-13

**Authors:** Judith U. Oguzie, Daniel B. Cummings, John T. Groves, Alex G. Hagan, Jessica Rodriguez, Gustavo Hernandez-Vidal, Gustavo Moreno-Degollado, Ismaila Shittu, Lyudmyla V. Marushchak, Thang Nguyen-Tien, Claudia M. Trujillo-Vargas, Diego B. Silva, Feng Li, John T. Richeson, Nicholas E. Schneider, Gregory C. Gray

**Affiliations:** ^1^ Division of Infectious Diseases, Department of Internal Medicine, University of Texas Medical Branch, Galveston, Texas, USA, utmb.edu; ^2^ Heritage Vet Partners, Madisonville, Tennessee, USA; ^3^ Livestock Veterinary Service, Eldon, Missouri, USA; ^4^ Ironsides Animal Health, Shelbyville, Kentucky, USA; ^5^ Universidad Autónoma de Nuevo León, Faculty of Veterinary Medicine, Escobedo, Nuevo León, Mexico, uanl.mx; ^6^ Department of Veterinary Science, University of Kentucky, Lexington, Kentucky, USA, uky.edu; ^7^ Department of Agricultural Sciences, West Texas A&M University, Canyon, Texas, USA, wtamu.edu; ^8^ Schneider Veterinary Services, Milliken, Colorado, USA; ^9^ Department of Microbiology and Immunology, University of Texas Medical Branch, Galveston, Texas, USA, utmb.edu; ^10^ Institute for Human Infections and Immunity, University of Texas Medical Branch, Galveston, Texas, USA, utmb.edu

**Keywords:** bovine respiratory disease, cattle farms, metagenomics, one health, respiratory viruses, zoonoses

## Abstract

Respiratory virus infections in cattle cause an estimated more than $1 billion in production losses and can threaten human health. During February 2024 to May 2025, we employed a One Health approach to surveil for respiratory viruses among cattle, farm workers, and environmental samples from 11 US and Mexican beef or dairy cattle farms. We studied nasal and ocular swabs from cattle, nasal swabs from cattle workers, bioaerosol samples, and other environmental farm samples using molecular and virological techniques. Among 26 distinct viruses identified in cattle, we detected bovine nidovirus 1, influenza D virus (D/OK‐like and D/660‐like), bovine coronavirus, bovine rhinitis A and B viruses, bovine respirovirus 3 and bovine respiratory syncytial virus (BRSV); 11 of the 26 detected viruses were non‐bovine‐associated. Two bovine rhinitis A virus was markedly divergent (provisionally designated BRAV‐4). Environmental metagenomics additionally identified influenza D virus, bovine coronavirus, and bovine rhinitis B virus. One human nasal swab tested positive for SARS‐CoV‐2 (cladeLF.7.3). Our findings reveal the presence of emerging, co‐circulating, and environmentally linked pathogens at the human–animal–environment interface, underscoring the constant need for One Health surveillance to safeguard livestock and mitigate zoonotic risk.

## 1. Introduction

Multiple respiratory pathogens contribute to cattle morbidity and mortality, leading to reduced production and increased costs [1]. Some of these pathogens may be zoonotic, placing cattle workers at risk of disease [2]. One of the largest cattle disease concerns, bovine respiratory disease complex (BRDC), is estimated to cost the US cattle industry more than $1 billion annually [3]. BRDC is a complex multifactorial syndrome caused by the interaction of multiple pathogens, host immune responses, and environmental stressors [4, 5]. While our understanding of the causative agents for BRDC has evolved over time, determining the most important etiologies remains challenging. Initially, BRDC was thought to be solely caused by bacterial infections; however, it is now globally recognized to be a polymicrobial disease [3, 6]. Currently, the mechanism for BRDC infection is thought to be initiated by a viral infection that damages the epithelium of the respiratory tract, leaving it susceptible to bacterial colonization and subsequent secondary infections [3]. Pathogens historically associated with BRDC include bacterial infections with *Manheimia haemolytica*, *Pasteurella multocida*, *Histophilus somni*, *or Mycoplasma bovis* and viral infections with bovine alphaherpesvirus 1 (BHV‐1), bovine viral diarrhea virus 1 (BVDV‐1), bovine respirovirus 3, and/or bovine respiratory syncytial virus (BRSV) [3, 6]. Specifically, the causes of BRDC are worthy of study not only for production reasons but also to avert zoonotic pathogen transmission.

In recent years, we have become increasingly aware of how cattle might amplify the zoonotic threats of influenza D virus (IDV) and avian influenza viruses [7, 8] to humans. IDV is a relatively newly recognized cause of BRDC [9]. Considerable evidence has shown the presence of antibodies to IDV in farm workers and general populations, indicating likely subclinical infection with the pathogen [7, 10, 11]. We published a seroepidemiological study of cattle workers in 2016, which documented that 97% of cattle workers in Florida had neutralizing antibodies to IDV compared to 18% among non‐cattle‐exposed control populations [10]. In 2023, we reported a 5‐day study of Colorado dairy workers in Colorado where we found 67% of 31 workers had molecular evidence of IDV in their nasal washes [12]. Recently, a scientific team reported serological evidence that IDV had likely caused subclinical infections in 73% of 612 study participants (96.7% among those with respiratory symptoms) in Northeast China [11].

The recent unanticipated spillover of highly pathogenic avian influenza (HPAI) H5N1 among US dairy cattle in early 2024 underscored our poor understanding of respiratory virus infections among cattle and the subsequent risk of zoonotic transmission to humans [13]. As of October 2025, a total of nearly 1100 US dairy farms [14] and at least 70 humans [15] have been documented to have evidence of H5N1 infection. Most human infections have been associated with dairy farm or poultry farm exposures. These observations strongly support the need for surveillance for pathogens causing illness in livestock, with the goal of reducing infections in both livestock and humans.

This study sought to supplement our current One Health molecular surveillance for pre‐pandemic pathogens on livestock farms with metagenomic sequencing to characterize viruses that might cause respiratory disease in cattle. Here, we present preliminary surveillance data from this ongoing study, conducted in collaboration with livestock veterinarians, supporting beef and dairy farms in Indiana, Kentucky, and Texas in the United States and Nuevo León, Mexico.

## 2. Methods

### 2.1. Study Site Enrollment and Sampling Protocol

A prospective One Health approach was used to surveil for respiratory viruses in a convenience sample of 11 cattle farms in Indiana (*n* = 1), Kentucky (*n* = 4), Texas (*n* = 3), and Mexico (*n* = 3) during 2024 and 2025. The farm’s cattle, workers, and environment were sampled and examined for the presence of novel respiratory viruses. The farms were visited upon enrollment and at 3–4‐month intervals. Informed consent and questionnaire data were collected from the farms and their workers.

The University of Texas Medical Branch (UTMB) Institutional Review Board approved the protocol (IRB # 23–0085). The animal sampling component was considered exempt by the UTMB Institutional Animal Care and Use Committee (March 13, 2023 review). All procedures were performed in compliance with applicable animal welfare and ethical guidelines. Additionally, all the methods were reported in accordance with the ARRIVE guidelines.

### 2.2. Sample Preparation and Processing

#### 2.2.1. Sample Collection

Samples were collected at each visit, including nasal/ocular swabs from cattle, nasopharyngeal (NP) swabs from farmworkers, swabs of the environment (tires, water point, trough, etc.), bioaerosol samples of areas where cattle and workers mix, and swabs from various dead animals found on the farm. Between scheduled visits, farm workers were encouraged to identify, collect, and ship swabs from cattle or enrolled workers who exhibited signs of respiratory symptoms using postage‐paid sample kits provided. All specimen swabs were collected in 2 mL of viral transport medium for preservation. Aerosol sampling was collected using the National Institute of Occupational Safety and Health (NIOSH) bioaerosol cyclone samplers (Tisch Environmental Inc., Cleves, OH), as previously described [16]. All samples were kept in an insulated cooler for transport.

All samples were processed according to standard operating procedures and aliquoted into multiple cryovials. RNA extraction for molecular analysis was performed using the QIAamp Viral RNA Mini Kit on the QIAcube Connect automated system (QIAGEN, Inc., Valencia, CA) per the manufacturer’s instructions. The RNA was eluted in 60 µl of AVE buffer and stored at −80°C.

#### 2.2.2. Polymerase Chain Reaction

Reverse transcription quantitative real‐time polymerase chain reaction (RT‐qPCR) targeting the matrix (M) gene of the influenza A virus (IAV) [17] was performed on the extracted RNA using the SuperScript III Platinum One‐Step RT‐qPCR System with Platinum Taq DNA Polymerase (Thermo Fisher Scientific, Inc., Waltham, MA). Any IAV‐positive cattle specimens were subtyped via H5‐specific RT‐qPCR and RT‐PCR assays for hemagglutinin (*HA*), neuraminidase (*NA*), and *HA* cleavage site, while IAV‐positive human specimens were subtyped for H1N1 [17]. Additionally, the RT‐qPCR assay, targeting the nucleoprotein gene of IDV [18], was performed on the extracted RNA using the AgPath‐ID One‐Step RT‐PCR Kit. Samples assessed via RT‐qPCR were considered positive when the cycle threshold (Ct) value was less than 40.

Specimens were also assessed by conventional RT‐PCR with a pan‐species coronavirus assay [19] to detect and characterize both previously recognized and novel human or animal coronaviruses. RT‐PCR amplicons were assessed by electrophoresis, and those with signals for the expected targeted molecular weights were then sent to Azenta Life Sciences (South Plainfield, NJ) for Sanger sequencing.

#### 2.2.3. Cell Culture Procedure

To isolate IDV from RT‐qPCR‐positive swab samples with (cycle threshold (Ct) values below 35), we used a swine testicular (ST) cell line (ATCC, cat. No. CRL‐1746), as previously described [20]. Briefly, ST cells were grown in high‐glucose Dulbecco’s Modified Eagle medium (DMEM; Thermo Fisher Scientific) supplemented with 10% (v/v) fetal bovine serum (Thermo Fisher Scientific) and 1x (v/v) antibiotic‐antimycotic solution (Thermo Fisher Scientific).

Once the cells reached 75%–85% confluency in 6‐well plates, they were washed with Dulbecco’s Phosphate‐Buffered Saline (Corning). A 20% (v/v) inoculum was prepared using a serum‐free maintenance medium comprising DMEM supplemented with 1x antibiotic‐antimycotic, 0.1% bovine serum albumin (Thermo Fisher Scientific), HEPES buffer (Thermo Fisher Scientific), 1 mM sodium pyruvate (Sigma–Aldrich), and 2 µg/mL TPCK‐treated trypsin (Sigma, cat. No. 4352157‐1KT). For inoculation, a 1 mL volume of the inoculum was transferred onto the ST monolayer and incubated for 1 h at 37°C in a 5% CO_2_ incubator to allow viral adsorption.

Following adsorption, the cells were washed with DPBS and overlaid with 3 mL of fresh maintenance medium. The cultures were incubated at 37°C in a 5% CO_2_ atmosphere. At 4–5 days post‐infection (dpi), supernatants were collected and replaced with 1 mL of fresh maintenance medium. The final harvest was performed at 7 dpi, following one freeze–thaw cycle.

The harvests were treated with TRIzol LS reagent (Invitrogen, Waltham, MA). The extracted RNA was stored at −80°C for further analysis. Virus isolation from samples collected in Mexico was conducted under enhanced biosafety level 3 (BSL3E) conditions, while samples from the United States were processed under enhanced biosafety level 2 (BSL2E) conditions at the UTMB.

### 2.3. Metagenomic Sequencing

We employed two approaches for metagenomic sequencing. First, we used the NEBNext Ultra II RNA Library Prep Kit (New England Biolabs, Ipswich, MA) to generate cDNA libraries, following the manufacturer’s protocol for Illumina sequencing. Second, we implemented a direct virus detection protocol for clinical specimens [21]. In this approach, extracted RNA underwent TurboDNase treatment to remove the host DNA, followed by reverse transcription into cDNA using random primers. Sequencing libraries were then constructed using the Illumina Nextera XT Library Prep Kit. The sequencing was performed on the Illumina NovaSeq X platform or the Element Biosciences AVITI Sequencer, generating 75 bp paired‐end reads on the NovaSeq X and 150 bp paired‐end reads on the AVITI platform.

### 2.4. Quality Control

Robust controls were applied across extraction, amplification, and sequencing to validate nucleic acid recovery and monitor contamination. Negative extraction controls (NECs) were included in every extraction batch; all NECs remained negative in subsequent downstream assays, indicating no contamination during the extraction phase. For RT‐PCR and RT‐qPCR, each assay plate contained positive controls (PCs) and no‐template controls (NTCs). No NTC was amplified on any plate, confirming that the PCR reagents and workspaces were contamination‐free.

For metagenomic sequencing, we implemented strict wet lab and computational safeguards. NECs and fresh negative controls were carried along throughout library preparation and sequencing to detect any downstream cross‐contamination. Libraries were prepared with unique dual indexes (UDIs) to minimize index hopping. Bioinformatic processing followed a stringent workflow with an a priori NTC threshold for viral read detection: any taxon detected in water controls was discarded, and a virus was considered a true positive only when its read count in a sample clearly exceeded the batch‐matched NTC background. In addition, we reported viruses only when at least 10% of the genome was assembled. Together, these measures reduced the risk of false positives and ensured the integrity of our results.

### 2.5. Bioinformatics Analysis

Raw FASTQ files from the sequencing runs were uploaded to the Chan Zuckerberg ID (CZ ID) platform (https://czid.org) for metagenomic analysis. Prior to uploading, metadata, including sample type, collection date, and location, were annotated according to the CZ ID metadata guidelines.

The CZ ID computational pipeline performed quality control by removing low‐quality reads and adapter sequences. Host‐derived reads were filtered out using Bowtie 2, and the remaining reads were aligned to the NCBI nucleotide (nt) reference database for taxonomic classification. The platform’s automated classification module identified bacterial, viral, fungal, and parasitic species, with taxonomic abundance visualized through interactive dashboards. For this study, we report only viral taxa for which at least 10 percent of the genome was assembled.

### 2.6. Phylogenetic Analysis

For IDV, we included the hemagglutinin‐esterase fusion (HEF) reference sequences for IDV lineages, along with our sequences and representative sequences obtained from various locations, which were downloaded from the NCBI GenBank database. We performed phylogenetic analyses for BRAV, BRBV, and bovine nidovirus by comparing our genomes with all complete or near‐complete sequences available in the NCBI. We compared our sequences with a curated panel of representative and reference genomes for all other viruses. To perform a multiple sequence alignment, we utilized MAFFT [22]. Subsequently, we constructed a maximum likelihood phylogenetic tree using IQ‐TREE [23] with 1000 bootstrap replicates. Trees were annotated on FigTree v1.4.4 (http://tree.bio.ed.ac.uk/software/figtree/).

## 3. Results

### 3.1. Overview of Sample Collection

**Figure 1:**
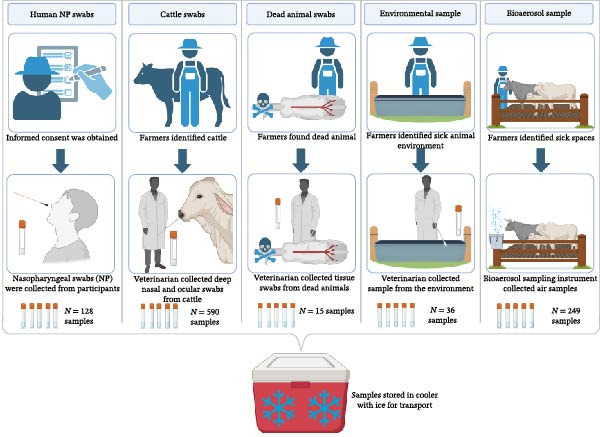
Farm sampling methods. This figure depicts the methods used to collect farm samples including human nasopharyngeal (NP) swabs, cattle swabs, dead animal swabs, environmental samples, and bioaerosol samples. The total number (N) of samples collected across all farms is enumerated for each sample type at the bottom of the figure. All samples were stored in an insulated cooler with ice for transport to the laboratory. This figure was created with BioRender.com.

Figure 1 provides an illustration of the samples collected at each farm from February 2024 to May 2025, including human nasal, cattle nasal/ocular, dead animal, environmental, and bioaerosol samples. Additionally, an overview of samples collected at each farm across all visits for the four locations is presented in Table 1. Briefly, from a single beef cattle farm in Indiana, 171 samples were collected, including 83 cattle nasal swabs, five ocular discharge swabs, four environmental water samples, 37 human nasal swabs, and 42 bioaerosol samples. A total of 412 samples were collected from four beef cattle farms in Kentucky, including 224 cattle nasal swabs, 13 tissue swabs from dead animals, 31 environmental samples, 30 human nasal swabs, and 114 bioaerosol samples. A total of 383 samples were collected from three dairy or beef cattle farms in Texas, including 238 nasal swabs, 2 swabs from a single dead animal, 1 environmental sample, 61 human nasal swabs, and 81 bioaerosol samples. In Mexico, 52 samples were collected on three beef cattle farms, comprising 40 cattle nasal swabs and 12 bioaerosol samples. Below, we further discuss our findings by viral assay, specimen source, and virus type.

**Table 1:** Counts and percentage of samples collected in each farm by state.

Farms by state	Cattle nasal swabs	Cattle ocular swabs	Dead animals swabs	Environmental samples	Human NP^a^ swabs	Bioaerosols
Indiana	*N* = 83	*N* = 5	*N* = 0	*N* = 4	*N* = 37	*N* = 42
F01	83 (100.0%)	5 (100.0%)	0 (0.0%)	4 (100.0%)	37 (100.0%)	42 (100.0%)
Kentucky	*N* = 224	*N* = 0	*N* = 13	*N* = 31	*N* = 30	*N* = 114
F01	80 (35.7%)	0 (0.0%)	1 (7.7%)	11 (35.5%)	12 (40.0%)	36 (31.6%)
F02	60 (26.8%)	0 (0.0%)	0 (0.0%)	7 (22.6%)	8 (26.7%)	33 (28.9%)
F03	72 (32.1%)	0 (0.0%)	12 (92.3%)	13 (41.9%)	10 (33.3%)	45 (39.5%)
F04	12 (5.4%)	0 (0.0%)	0 (0.0%)	0 (0.0%)	0 (0.0%)	0 (0.0%)
Texas	*N* = 238	*N* = 0	*N* = 2	*N* = 1	*N* = 61	*N* = 81
F01	60 (25.2%)	0 (0.0%)	0 (0.0%)	0 (0.0%)	36 (59.0%)	33 (40.7%)
F02	79 (33.2%)	0 (0.0%)	2 (100.0%)	1 (100.0%)	25 (41.0%)	48 (59.3%)
F03	99 (41.6%)	0 (0.0%)	0 (0.0%)	0 (0.0%)	0 (0.0%)	0 (0.0%)
Mexico	*N* = 40	*N* = 0	*N* = 0	*N* = 0	*N* = 0	*N* = 12
F00	4 (10.0%)	0 (0.0%)	0 (0.0%)	0 (0.0%)	0 (0.0%)	0 (0.0%)
F03	20 (50.0%)	0 (0.0%)	0 (0.0%)	0 (0.0%)	0 (0.0%)	12 (100.0%)
F04	16 (40.0%)	0 (0.0%)	0 (0.0%)	0 (0.0%)	0 (0.0%)	0 (0.0%)

^a^Human nasopharyngeal (NP) swabs.

### 3.2. Influenza D Virus, Pan‐Coronavirus, and Influenza A Virus Molecular Assay Results

**Figure 2:**
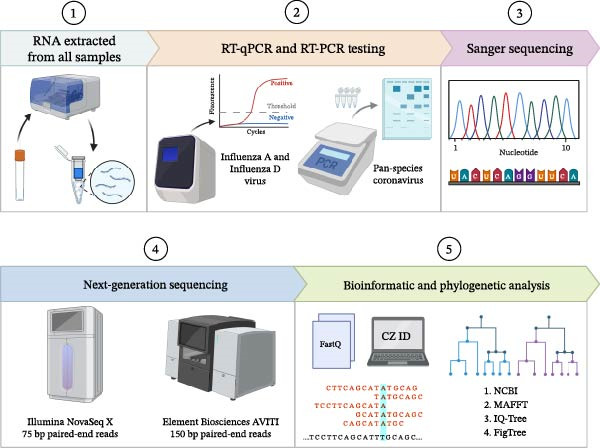
Testing methods. This figure outlines the workflow implemented to identify and characterize viral presence in all the samples collected. Testing methods implemented included RNA extraction, RT‐qPCR and RT‐PCR, Sanger Sequencing, Next Generation Sequencing, Bioinformatic and Phylogenetic analysis. This figure was created with BioRender.com.

Following RNA extraction, we performed RT‐qPCR for IAV and IDV and RT‐PCR using a pan‐coronavirus assay. Pan‐coronavirus–positive amplicons were Sanger‐sequenced, as detailed in Figure 2. In Indiana, molecular evidence of IDV was identified in eight of 171 (4.7%) samples comprising five cattle nasal swabs and three environmental water samples (Table 2). In Kentucky, 39 out of 412 (9.5%) samples had molecular evidence of IDV, including 33 cattle nasal swabs, one dead cow tracheal swab, and five environmental samples from water troughs (Table 2). In Texas, 37 out of 238 (15.5%) cattle nasal swab samples had molecular evidence of IDV. In Mexico, 17 out of 40 (42.5%) cattle nasal swabs had molecular evidence of IDV, with five of 12 (41.7%) bioaerosol samples having high Ct values (35.5–37.9) (Supporting Information 1: Table S1). We successfully isolated 25 IDV strains from the IDV‐positive samples using ST cells.

**Table 2:** Summary of total samples collected and positive results for each molecular test stratified by state.

Sample type	Indiana				Kentucky			Texas			Mexico		
	Total	IDV (+)^a^	Pan‐CoV(+)^b^	IAV (+)^c^	Total	IDV (+)	Pan‐CoV (+)	Total	IDV (+)	Pan‐CoV (+)	Total	IDV (+)	Pan‐CoV (+)
Cattle nasal swabs	83	5 (6.0%)	37 (44.6%)	0 (0.0%)	224	33 (14.7%)	53 (23.7%)	238	37 (15.5%)	61 (25.7%)	40	17 (42.5%)	9 (22.5%)
Cattle ocular swabs	5	0 (0.0%)	2 (40.0%)	0 (0.0%)	0	0 (0.0%)	0 (0.0%)	0	0 (0.0%)	0 (0.0%)	0	0 (0.0%)	0 (0.0%)
Dead animals	0	0 (0.0%)	0 (0.0%)	0 (0.0%)	13	1 (7.7%)	0 (0.0%)	2	0 (0.0%)	0 (0.0%)	0	0 (0.0%)	0 (0.0%)
Environmental samples	4	3 (75.0%)	0 (0.0%)	0 (0.0%)	31	5 (16.1%)	2 (6.5%)	1	0 (0.0%)	0 (0.0%)	0	0 (0.0%)	0 (0.0%)
Human NP swabs	37	0 (0.0%)	0 (0.0%)	1 (2.7%)	30	0 (0.0%)	1 (3.3%)	61	0 (0.0%)	0 (0.0%)	0	0 (0.0%)	0 (0.0%)
Bioaerosols	42	0 (0.0%)	0 (0.0%)	0 (0.0%)	114	0 (0.0%)	0 (0.0%)	81	0 (0.0%)	0 (0.0%)	12	5 (41.7%)	2 (16.7%)
Total	171	8 (4.7%)	39 (22.8%)	1 (0.6%)	412	39 (9.5%)	56 (13.6%)	383	37 (9.6%)	61 (15.9%)	52	22 (42.3%)	11 (21.2%)

^a^Influenza D virus (IDV) RT‐qPCR positive results.

^b^Pan‐species coronavirus (Pan‐CoV) RT‐PCR positive results.

^c^Influenza A (IAV) RT‐qPCR positive results.

In Indiana, 39 (22.8%) specimens were positive by the pan‐coronavirus assay, consisting of 37 cattle nasal and two cattle ocular swabs (Table 2). Of those characterized via Sanger sequencing with good quality scores, 22 cattle nasal and 2 cattle ocular swabs were identified as bovine coronavirus (BCoV), and one rodent coronavirus (RCoV) was identified from a cattle nasal swab. In Kentucky, 56 (13.6%) samples were similarly positive: 53 from cattle nasal swabs, two environmental water samples, and one human nasal swab. Of these, 30 cattle nasal swabs and 1 environmental swab were identified as BCoV, while the second environmental swab was identified as RCoV. In Texas, 61 out of 238 (25.7%) cattle nasal swabs had molecular evidence of a coronavirus; from these, we were able to characterize 38 as bovine coronaviruses. One SARS‐CoV‐2 virus was also identified from a cattle nasal swab in Texas, as we had previously reported [16]. In Mexico, nine out of 40 (22.5%) cattle nasal swabs and two out of 12 (16.7%) bioaerosol samples had molecular evidence of a coronavirus. Of these, four cattle nasal swabs were identified as BCoV, while five other cattle nasal swabs and one bioaerosol sample were characterized as RCoV. The RCoV detections were previously reported [24].

No specimen from Kentucky or Mexico had molecular evidence of IAV. One human nasal swab from Indiana was positive for IAV (Ct = 32.47) and was confirmed as H1N1 (Ct = 31.20) (Supporting Information, Section 1: Table S1). IAV was also detected in samples collected from dairy cattle in Texas during early 2024, which was previously reported [16].

### 3.3. Co‐Infectivity Profile by RT‐PCR

Several studies have documented evidence of co‐infection with IDV and BCoV using molecular and sequencing‐based methods [25, 26]. Within all the samples collected, two (2.4%) cattle nasal swabs, from the single farm in Indiana, showed coinfection with both IDV and BCoV (Supporting Information, 1: Table S1). Twelve (5.3%) cattle nasal swabs from two different farms in Kentucky and one (3.2%) environmental water swab showed coinfection with both IDV and pan‐coronavirus (9 characterized as BCoV) (Supporting Information 1: Table S1). In one Texas farm, 18 (7.6%) cattle nasal swab samples showed coinfection with both IDV and pan‐coronavirus (7 characterized as BCoV) (Supporting Information, 1: Table S1). Additionally, six (15.0%) cattle nasal swabs from two different farms in Mexico and one (8.3%) bioaerosol sample exhibited coinfection with both IDV and pan‐coronavirus (3 characterized as BCoV and 3 characterized as RCoV) (Supporting Information 1: Table S1).

### 3.4. Metagenomics Next‐Generation Sequencing Overview

Following molecular screening, 84 samples were selected for next‐generation sequencing (NGS) based on IDV detection, molecular detection of coronavirus via our pan‐coronavirus assay, and representation across farms, sampling sites, and specimen types.

Our analysis revealed the presence of several viruses associated with BRDC (Supporting Information, 2: Table S2) and several non‐bovine‐associated viruses. In total, we detected 26 viruses, representing 16 different viral families.

### 3.5. Genomic Characterization and Detection of IDV Lineages D/OK and D/660

**Figure 3:**
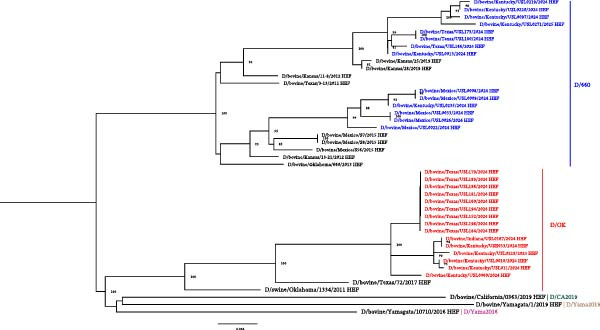
Maximum Likelihood phylogenetic tree of the hemagglutinin‐esterase fusion (HEF) gene of studied influenza D viruses. Sequences generated in this study are highlighted in blue (D/660 lineage) and red (D/OK lineage). Multiple sequence alignment was performed using MAFFT, and the tree was generated using IQ‐TREE. Reference strains representing major influenza D virus lineages (black text) are included. The scale bar indicates the number of nucleotide substitutions per site.

IDV is a recognized orthomyxovirus primarily infecting cattle, potentially spreading to other livestock species [27, 28]. In this study, we successfully sequenced 55 samples that had molecular evidence of IDV. Among these, we detected IDV reads in several specimens and recovered partial‐to‐near‐complete genome coverage (14%–100%) in 39 specimens. Phylogenetic analysis revealed that 29 samples with complete or nearly complete HEF gene sequences clustered within both the D/OK and D/660 lineages of IDV (Figure 3).

The D/OK lineage is one of the two primary genetic lineages of IDV, while it was first identified in a diseased pig with influenza illness (*D/swine/Oklahoma/1334/2011*), it is now predominantly found in cattle [20, 29]. This lineage has been reported globally and is the most prevalent IDV lineage in the United States and Mexico [29, 30]. It is characterized by genetic stability and widespread circulation in cattle populations, with evidence suggesting its contribution to the BRDC, a significant economic burden for the cattle industry [29, 30].

Variations in the HEF gene, which plays a key role in viral attachment and entry, have been observed within the D/OK lineage, facilitating efficient host adaptation [31]. Although primarily associated with cattle, the D/OK lineage has also been detected in swine and small ruminants, and the presence of human antibodies has been documented, raising concerns about its zoonotic potential [12, 20, 32].

We also detected sequences clustering within the D/660 lineage, initially identified in *D/bovine/Oklahoma/660/2013*. D/OK once dominated in Europe and Asia, but with the emergence of D/660, both clades now cocirculate in the United States, Europe, and Asia. New clades, D/Yama2016 and D/Yama2019 were first identified in Japan, with subsequent detection of D/Yama2019 in China [29]. The detection of D/660 in our samples suggests that multiple IDV lineages may be cocirculating in cattle populations in this region, potentially enhancing opportunities for viral reassortment and increased genetic diversity.

Detecting both D/OK and D/660 lineages underscores the genetic complexity of IDV circulating in livestock and emphasizes the importance of ongoing research to understand the lineage‐specific pathogenicity and transmission dynamics.

### 3.6. SARS‐CoV‐2 in a Human Nasal Swab

Of the 128 human nasal swabs collected from all farms, only 1 nasal swab from Kentucky was positive for the pan‐coronavirus assay; all other samples were negative across all assays tested. This nasal swab sample was collected from a 35‐year‐old male who presented with symptoms of respiratory illness, including a scratchy throat. The positive sample was further characterized as SARS‐CoV‐2 using the Sanger sequencing method. We performed NGS via a metagenomic approach to gain deeper genomic insights. Genome assembly yielded a SARS‐CoV‐2 genome with 99.5% coverage (GenBank accession number PX474634). Phylogenetic analysis classified the virus within the recently identified 24H clade, which is an omicron sublineage (LF.7.3.1). This clade harbors distinct genetic mutations that may influence viral transmissibility, immune evasion, or pathogenicity [33]. Although this finding is not thought to be associated with zoonotic transmission, the emergence of this clade underscores the ongoing evolution of SARS‐CoV‐2.

### 3.7. Respiratory Viruses in Environmental Samples

Environmental samples collected from farm 3 in Kentucky revealed the presence of multiple respiratory viruses with potential implications for livestock health and zoonotic spillover. Molecular testing indicated that one environmental sample collected from a water sinkhole was positive for coronavirus, while another sample collected from a water swab (Pen 4) was positive for both pan‐coronavirus and IDV.

In the water swab collected from the sinkhole in Pen 2, coronavirus was detected via a pan‐coronavirus PCR assay and confirmed to be a RCoV by Sanger sequencing. While metagenomic sequencing identified reads, the genome coverage was insufficient for genome assembly. This finding underscores the potential role of rodents as reservoirs of pathogens, including coronaviruses, that can be transmitted into shared environments with livestock. Additionally, we achieved 93% genome coverage for a virus in the *Nodaviridae* family and 45.5% coverage for Wenzhou noda‐like virus 1. Detecting these non‐bovine viruses in a livestock‐associated environment highlights the complexity of viral communities at the human–animal–environment interface.

The environmental sample for the water swab in Pen 4 tested positive for IDV (Ct = 29.69) and a coronavirus (Supporting Information 1: Table S1). IDV contigs were identified from this specimen; however, only partial genomes could be assembled. Detection of IDV nucleic acids in environmental water is consistent with environmental contamination and warrants further investigation into potential persistence and indirect exposure pathways; however, molecular detection alone does not establish viral viability or transmission. We also assembled a near‐complete genome of bovine rhinitis B virus (BRBV) from this sample. Phylogenetic analysis revealed that the virus detected in the environmental specimen clustered with bovine nasal swab specimens collected on the same farm, all belonging to BRBV‐5. These findings suggest potential environmental contamination originating from the bovine host. Although a partial genome of BCoV was recovered, it was unsuitable for downstream phylogenetic analysis. The detection of multiple bovine respiratory viruses in environmental water sources could indicate viral persistence and risk for inter‐animal transmission within confined farm settings.

In addition, we examined one bioaerosol sample from a farm in Mexico that had molecular evidence for IDV (high Ct value of 35.4) (Supporting Information 1: Table S1) as well as bovine rotavirus C and bovine rhinitis A (BRAV) and B virus genomes (Supporting Information 2: Table S2). Detecting respiratory and enteric viral nucleic acids in airborne particles indicates that viral genetic material can be aerosolized and deposited onto surfaces, which may create opportunities for environmental exposure (airborne or fomite‐mediated), particularly in enclosed or poorly ventilated animal housing environments. However, detection by molecular methods does not demonstrate the presence of an infectious virus or confirm transmission.

Collectively, these findings emphasize the importance of environmental surveillance in agricultural settings for the early detection of pathogens of veterinary and public health importance. Environmental reservoirs, such as water and bioaerosols, may contribute to maintaining and disseminating respiratory viruses, including those with zoonotic potential.

### 3.8. Bovine Coronavirus and Bovine Nidovirus 1 Detection From Bovine Nasal and Ocular Swab Specimens

**Figure 4:**
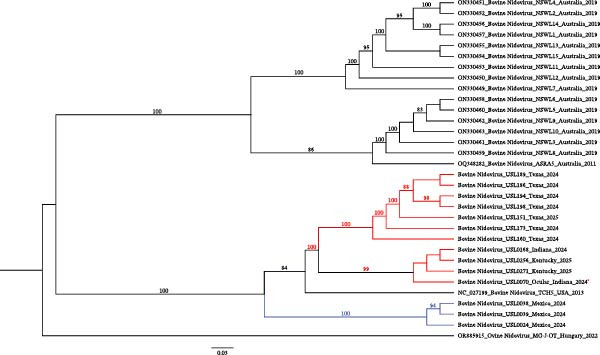
Maximum‐likelihood phylogeny of bovine nidovirus 1. Maximum‐likelihood tree inferred from nucleotide sequences of bovine nidovirus, midpoint rooted. The ocular sample is indicated with a red star. Sequences from the USA are shown in red, and those from Mexico in blue. Bootstrap support values are shown for key branches.

From a beef cow’s ocular swab in Indiana, we successfully assembled a BCoV genome, along with a near‐complete genome of bovine nidovirus 1 (BoNV‐1). While BCoV has been reported in ocular swabs from cattle [34, 35] and BoNV‐1 is typically detected in NP samples [36], to our knowledge, this is the first documented detection of BoNV‐1 in an ocular swab. The BCoV genome shared a 99.82% nucleotide identity with strain OP037441.1, isolated in 2022 from a bovine fecal sample, while the BoNV‐1 genome showed 95.13% identity with reference strain NC_027199.1, identified in 2013 from a bovine nasal swab. Phylogenetic analysis of the BoNV‐1 genome from ocular discharge revealed site‐specific clustering by farm/location (Figure 4).

The detection of novel BoNV‐1 from an ocular site highlights the need to consider alternative mucosal surfaces in surveillance efforts for respiratory pathogens in cattle. It also raises important questions about the tissue tropism and potential transmission routes of BoNV‐1, warranting further investigation into its pathogenicity and epidemiological significance.

### 3.9. Bovine Coronavirus in Cattle Nasal Swabs

Metagenomic analysis of bovine nasal swab samples detected BCoV contigs in 39 specimens, of which 19 represented complete or near‐complete genomes. Phylogenetic analysis showed that most sequences (*n* = 17) clustered with strain OP037441.1, a genotype representative of the classical respiratory/fecal BCoV lineage detected in cattle fecal samples in 2022. The remaining sequences (*n* = 2) clustered with strain OP037398. These findings indicate a predominance of classical BCoV lineages among the sampled cattle, with limited evidence of divergent variants. The detection of two sequences clustering with OP037398, a distinct variant within the classical group, suggests cocirculation of closely related genotypes. Although both OP037441 and OP037398 belong to the classical respiratory/fecal lineage, their divergence points to ongoing viral diversification and possible adaptation within cattle populations.

### 3.10. Bovine Nidovirus 1 in Bovine Nasal Swab Samples

BoNV‐1 was detected in 19 nasal swab specimens, yielding 14 complete or near‐complete genomes. Phylogenetic analysis revealed two distinct clades (Figure 4). The first comprised specimens from farms in the United States, clustering with the only available U.S. reference sequence in the National Center for Biotechnology Information (NCBI) (NC_027199.1), originally described from a bovine respiratory specimen [37]. The second clade contained three sequences from Mexico. Although BoNV‐1 had been previously reported [9] in Mexico, no sequence data were available in NCBI. Here, we present the first complete genome sequences of the virus from Mexico, which form a distinct cluster in the phylogenetic tree. These findings indicate that BoNV‐1 is present in the cattle populations sampled.

### 3.11. Bovine Rhinitis A Virus and Bovine Rhinitis B Virus

**Figure 5:**
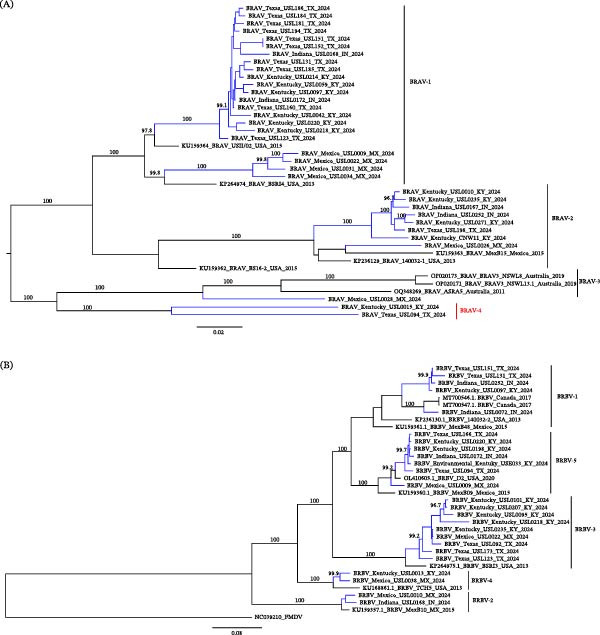
Bovine Rhinitis A Virus (BRAV) and Bovine Rhinitis B Virus (BRBV) Trees. Maximum likelihood phylogeny of BRAV (A) and BRBV (B) based on nucleotide sequences. Tree A is mid‐point rooted and Tree B is rooted to NC039210. Four distinct BRAV lineages are indicated in the annotation, with our samples highlighted in blue. The novel genotype, designated as BRAV‐4, is shown in red. Bootstrap values for major nodes are provided for both trees. Five distinct BRBV lineages are indicated in the annotation, with our samples highlighted in blue.

Previously, two genotypes of bovine rhinitis A virus were designated as BRAV‐1 and BRAV‐2, with a recent study identifying a distinct third genotype, BRAV‐3 [38]. In this study, we identified reads for the virus in 54 specimens, from which we assembled 34 complete or near‐complete BRAV genomes. Of these, 29 high‐quality genomes were retained for phylogenetic anlysis, encompassing all previously known genotypes: BRAV‐1 (*n* = 24), BRAV‐2 (*n* = 2), and BRAV‐3 (*n* = 1). In addition, we detected two BRAV sequences that were genetically distinct from genotypes 1–3. These two sequences, derived from nasal swabs collected from cattle in Kentucky and Texas, formed a separate, well‐defined cluster on the phylogenetic tree (Figure 5A). We refer to this cluster as a divergent BRAV lineage, provisionally designated BRAV‐4 (this study).

BLASTn analysis indicated that the two divergent BRAV‐4 sequences shared 85.52% and 85.53% nucleotide identity with KT948520.1 and 83.76% and 84.05% nucleotide identity with the BRAV reference genome NC_038303.1. BLASTx analysis showed that these sequences share 93.71%–93.74% amino acid identity with their closest hits (AKA20761 polyprotein). For BRAV‐3, BLASTx analysis demonstrated a 96.56% amino acid identity to the genotype previously reported in Australia [38]. Our findings represent the first detection of BRAV‐3 in both the United States and Mexico as well as the first report of BRAV‐4.

Detecting multiple BRAV genotypes, including a novel lineage, highlights this virus’s substantial genetic diversity and ongoing evolution in cattle populations. Different genotypes across geographically distant herds suggest potential regional adaptation, viral persistence, or independent introduction events. From an epidemiological perspective, such diversity may influence viral transmission dynamics, pathogenicity, and the effectiveness of diagnostic assays or future vaccines, underscoring the need for continued genomic surveillance to inform BRDC control strategies.

In addition, we detected BRBV sequences from 43 specimens, resulting in 17 complete to near‐complete genome assemblies. Our sequences clustered with all five known BRBV types (1–5). Specifically, eight sequences were identified as BRBV‐5, 3 as BRBV‐1, 2 as BRBV‐2, 2 as BRBV‐4, and 2 as BRBV‐3 (Figure 5B). Seven specimens were coinfected with BRAV and BRBV, with complete or near‐complete genomes for both viruses.

### 3.12. Bovine Enterovirus

In addition, contigs corresponding to enterovirus E4 were detected in 20 samples, including 11 complete or near‐complete genomes. The bovine enterovirus sequences identified in this study clustered within the EV‐E4 group (Figure 6A). BLASTn analysis showed that all sequences shared the highest nucleotide identity with the Enterovirus E4 reference strain KU172420.1, initially reported by Mitra et al. [9].

Figure 6(A–D) Bovine enterovirus phylogeny. Maximum‐likelihood tree (midpoint‐rooted) with bootstrap support shown at major nodes; branch lengths indicate substitutions per site. Study sequences (clustered with Enterovirus E4) are highlighted in red. (B) Bovine respirovirus 3 (BRV‐3) phylogeny and genotype assignments. Maximum‐likelihood tree rooted to NC_002161, inferred from two study sequences (red), contextual sequences, and representative Genotype A–C references from GenBank. The Mexico specimen falls within Genotype B, while the Texas specimen clusters with Genotype C. Branch lengths indicate the number of substitutions per site. (C) Bovine respiratory syncytial virus (BRSV) phylogeny and subgroup assignments. Maximum‐likelihood tree of the G‐gene nucleotide sequences (midpoint‐rooted), including two study sequences (red), contextual sequences, and subgroup representatives. Both study sequences cluster within Subgroup III. Branch lengths indicate substitutions per site. (D) Bovine viral diarrhea virus (BVDV) polyprotein phylogeny. Maximum‐likelihood tree (midpoint‐rooted) from nucleotide sequences of study genomes (red), BVDV‐1 subgenotype references, and sequences from NCBI. Both study sequences cluster within the BVDV‐1b group. Branch lengths indicate substitutions per site.

**Figure:**
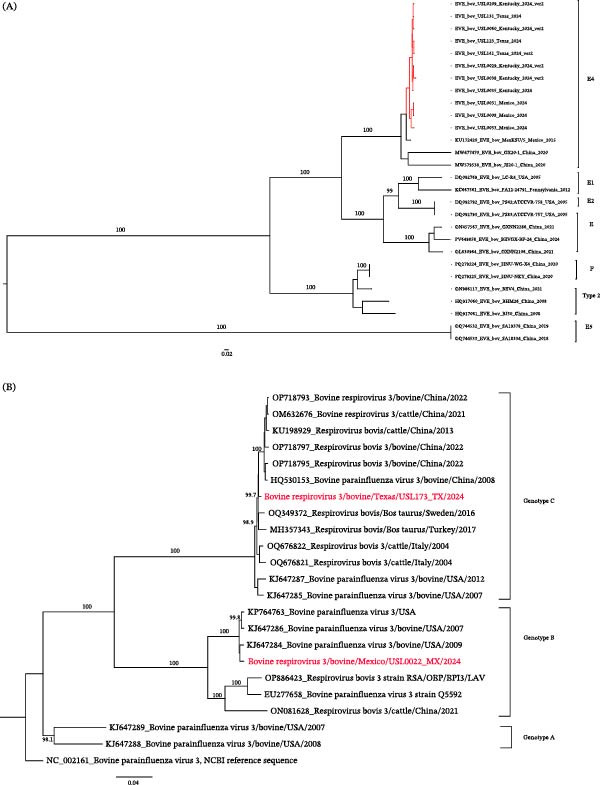

**Figure:**
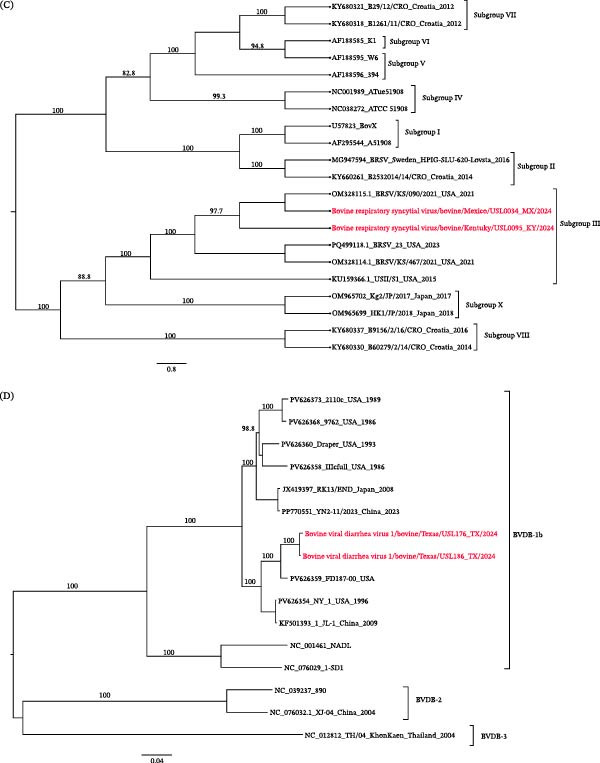

### 3.13. Bovine Respirovirus 3

We identified bovine respirovirus 3 (BRV3) in five bovine nasal swab samples. BRV3 is a major viral contributor to BRDC and upper respiratory tract infections [39, 40]. Phylogenetic analysis revealed that the sequences formed two distinct clusters corresponding to genotypes B and C (Figure 6B). One sample from Mexico showed 99.13% nucleotide identity with strain KJ647284.1, a genotype B virus previously reported in U.S. cattle [41]. Genotype B viruses are widely distributed in North America and have been frequently associated with respiratory disease outbreaks in feedlot cattle, reflecting the persistence of endemic lineages in U.S. herds. The Texas sample exhibited 99.14% identity with strain OQ349372.1, clustering within genotype C. Although genotype C viruses have been reported less frequently in U.S. cattle, they are increasingly detected worldwide, suggesting ongoing diversification and lineage intermixing [42].

Our data confirm the cocirculation of BRV3 genotypes B and C in the sampled cattle population, underscoring the genetic diversity of circulating strains and the need for continued genomic surveillance. To our knowledge and based on the NCBI database, BRV3 genotypes have not yet been reported from Mexican cattle; previous studies in Mexico documented exposure or PCR positivity without sequence‐based genotyping.

### 3.14. Bovine Respiratory Syncytial Virus (BRSV)

BRSV was detected in three nasal swab specimens from clinically ill cattle, and two near‐complete genomes were successfully assembled. BRSV is a cause of respiratory disease in young calves and a key contributor to BRDC [40]. Its ability to evade host immunity through antigenic variation underscores the importance of continuous genomic surveillance in informing vaccine development and guiding outbreak control strategies [43].

Phylogenetic analysis revealed that both genomes clustered within the BRSV subgroup III lineage (Figure 6C). Subgroup III is one of several molecular subgroups defined by sequence variation in the attachment glycoprotein gene and is widely reported in North America and Europe. This lineage has been implicated in severe BRDC outbreaks in feedlot and dairy operations, leading to considerable economic losses [44]. Our sequences showed the highest nucleotide identity (98.86%) to reference subgroup III strains, including KU159366.1 (USA, 2014). Detecting this lineage in our samples expands the known geographic distribution of subgroup III and provides additional genomic data for comparative analyses of its evolution and epidemiology.

### 3.15. Pestivirus Type 1 in Bovine Nasal Swabs

We detected reads consistent with pestivirus type 1 from our data, also known as bovine viral diarrhea virus 1 (BVDV‐1). Pestiviruses are members of the *Flaviviridae* family and are important cattle pathogens associated with respiratory disease, reproductive failure, and immunosuppression [45]. We assembled BVDV‐1 contigs from these samples in four specimens, including two nearly complete genomes that shared the highest nucleotide identity (≈97%) with sequence PV626359.1. Phylogenetic analysis showed that both near‐complete genomes clustered with PV626359.1, confirming their classification as BVDV‐1b. All recovered sequences belonged to the BVDV‐1b subgenotype (Figure 6D).

BVDV‐1b is the most prevalent subgenotype in North America [46]. It is associated with BRDC and frequently detected in beef and dairy cattle. Its widespread circulation in the U.S. carries important implications for cattle health, productivity, and vaccination strategies.

### 3.16. Bovine Rotaviruses and Bovine Torovirus

Metagenomic sequencing of bovine nasal swab samples revealed diverse rotaviruses, spanning multiple genogroups and gene segments. The identified sequences were classified into three major groups: rotavirus A (RVA), rotavirus B (RVB), and rotavirus C (RVC), highlighting the genetic heterogeneity of rotavirus circulating in cattle.

Additionally, bovine torovirus contigs and bovine picobirnavirus contigs were detected in nasal swab specimens. Bovine torovirus, a member of the family *Tobaniviridae*, is classically associated with enteric infections in calves but has been increasingly reported in respiratory samples [47].

### 3.17. Non‐Bovine Viruses in Bovine Nasal Swab Samples

Several non‐bovine‐associated viruses were detected, suggesting possible environmental contamination, dietary origins, or incidental carriage. In total, 12 distinct non‐bovine viruses were identified across multiple samples. The most frequently detected virus was a member of the *Nodaviridae* family found in seven samples. Other viruses detected in more than one sample included apis dicistrovirus, flumine dicistrovirus 40, and turnip vein‐clearing virus (each in two samples).

Viruses detected in single samples included wenzhou noda‐like virus, aphid lethal paralysis virus, medicago sativa alphapartitivirus, *Mitoviridae*, *Partitiviridae*, praha dicistro‐like virus, Shenzhen reo‐like virus, and *sobemovirus* sp. These viral taxa are typically associated with insects, plants, or fungi rather than cattle, and their presence may reflect environmental exposure or incidental cross‐contamination during sample collection.

### 3.18. Detection of Sapovirus in Rodent Fecal Specimens

Fecal swab specimens collected from dead rodents found on one of the farms in Kentucky yielded sequence reads consistent with sapovirus. Rodents are highly adaptable and prolific mammals that frequently inhabit areas near human dwellings and agricultural operations, increasing the risk of zoonotic pathogen transmission. Detecting sapovirus in rodent fecal swabs aligns with prior evidence that the virus is shed via the gastrointestinal tract [48]. This finding supports the potential for active enteric infection and viral excretion. The presence of sapovirus in rodent feces highlights the risk of environmental contamination, particularly in food production and storage settings.

## 4. Discussion

This study provides a metagenomic snapshot of respiratory viral pathogens circulating in cattle in the United States and Mexico. By leveraging high‐throughput sequencing, we identified a diverse range of viral taxa, many associated with the BRDC, a leading cause of morbidity and economic losses in the cattle industry [49]. One important finding was the widespread detection of IDV, particularly of the D/OK and D/660 lineages, across farms in all four regions. While the D/OK lineage has been historically dominant in North America [20], the D/660 lineage, first identified in the United States, has since gained global prevalence [25, 50–52].

The association between IDV and the BRDC complex is becoming increasingly recognized [27, 53], and its detection in both nasal and environmental water samples suggests potential indirect transmission routes and highlights the importance of environmental monitoring. The presence of these lineages across geographically diverse farms underscores their capacity for broad dissemination and their potential impact on cattle health and productivity. Additionally, there is growing evidence of IDV detection in humans [7, 10, 54, 55].

Beyond IDV, our dataset revealed considerable viral diversity. We report diverse BRAV and BRBV serotypes, including the recently proposed BRAV‐3, and identify a novel divergent BRBV‐4, which indicates ongoing viral adaptation that may affect cross‐protective immunity and complicate vaccine design. The detection of novel BoNV‐1 in an ocular swab expands the current understanding of its tissue tropism and highlights the need for broader surveillance protocols that include non‐traditional sample types.

Environmental samples also provided compelling evidence of virus circulation. Bioaerosols and water swabs showed evidence of IDV, BCoV, BRBV, and RCoV, demonstrating how farm environments can harbor multiple viruses with both veterinary and zoonotic significance.

## 5. Conclusion

Altogether, our supplemental metagenomic characterization of viral pathogens on livestock farms emphasize the complexity of respiratory virus dynamics in agricultural systems. Frequent co‐infections may exacerbate clinical signs and complicate diagnosis and treatment. The presence of non‐bovine viruses in cattle nasal swabs suggests possible contamination, dietary exposures, or novel host–virus interactions that merit further exploration. From a One Health perspective, these results underscore the importance of integrated surveillance efforts across livestock, humans, and shared environments. Early detection of emerging viral threats remains critical for protecting animal health and mitigating potential zoonotic spillovers.

## Author Contributions

Gregory C. Gray and Judith U. Oguzie conceived and designed the study. Judith U. Oguzie, Daniel B. Cummings, John T. Groves, Alex G. Hagan, Jessica Rodriguez, Gustavo Hernandez‐Vidal, Gustavo Moreno‐Degollado, Ismaila Shittu, Lyudmyla V. Marushchak, Thang Nguyen‐Tien, Claudia M. Trujillo‐Vargas, Diego B. Silva, Feng Li, John T. Richeson, Nicholas E. Schneider, and Gregory C. Gray developed the methodology and carried out the investigation. Judith U. Oguzie performed the formal analysis and curated the data together with Jessica Rodriguez, Ismaila Shittu, Lyudmyla V. Marushchak, Thang Nguyen‐Tien, Claudia M. Trujillo‐Vargas, and Diego B. Silva. Judith U. Oguzie and Gregory C. Gray drafted the original manuscript, and all authors contributed to writing, reviewing, and editing the manuscript. Judith U. Oguzie and Jessica Rodriguez prepared the visualizations. Gregory C. Gray supervised the study and obtained the funding.

## Funding

This project was supported by the Agriculture and Food Research Initiative Competitive Grant from the American Rescue Plan Act (award number 2023‐70432‐39558) through USDA APHIS, from USDA‐ARS Agreement 58‐3022‐4‐048, and from Professor Gregory C. Gray’s startup funding from UTMB. The findings and conclusions in this presentation are those of the authors and should not be construed to represent any official USDA or US government determination or policy.

## Disclosure

All authors have read and approved the final manuscript.

## Ethics Statement

All procedures were performed in compliance with applicable animal welfare and ethical guidelines. Additionally, all the methods were reported in accordance with the ARRIVE guidelines.

## Conflicts of Interest

The authors have no competing interests to disclose.

## Supporting Information

Additional supporting information can be found online in the Supporting Information section.

## Supporting information


**Supporting Information 1** Table S1. PCR test results and detailed sample‐level metadata for all specimens included in this study.

**Supporting Information 2** Table S2. Viruses detected by next‐generation sequencing (NGS) across all samples, including read metrics and genome coverage.

## Data Availability

The data that support the findings of this study are available from the corresponding author upon request. Genbank accession numbers PX485343‐PX485499, PX529686‐PX529734, PX529806‐PX529820, and PX680750‐PX680802.
